# TRPV1: A promising therapeutic target for skin aging and inflammatory skin diseases

**DOI:** 10.3389/fphar.2023.1037925

**Published:** 2023-02-15

**Authors:** Tengfei Xiao, Mingzhong Sun, Chuanxiang Zhao, Jingjing Kang

**Affiliations:** ^1^ Department of Clinical Laboratory, The Sixth Affiliated Hospital of Nantong University, Yancheng Third People’s Hospital, Yancheng, Jiangsu, China; ^2^ Institute of Medical Genetics and Reproductive Immunity, School of Medical Science and Laboratory Medicine, Jiangsu College of Nursing, Huai’an, Jiangsu, China; ^3^ Department of Clinical Laboratory, Affiliated Hospital of Nanjing University Medical School, Yancheng First People’s Hospital, Yancheng, Jiangsu, China

**Keywords:** TRPV1, Ca ^2+^, skin aging, inflammatory skin diseases, therapeutic strategy

## Abstract

TRPV1 is a non-selective channel receptor widely expressed in skin tissues, including keratinocytes, peripheral sensory nerve fibers and immune cells. It is activated by a variety of exogenous or endogenous inflammatory mediators, triggering neuropeptide release and neurogenic inflammatory response. Previous studies have shown that TRPV1 is closely related to the occurrence and/or development of skin aging and various chronic inflammatory skin diseases, such as psoriasis, atopic dermatitis, rosacea, herpes zoster, allergic contact dermatitis and prurigo nodularis. This review summarizes the structure of the TRPV1 channel and discusses the expression of TRPV1 in the skin as well as its role of TRPV1 in skin aging and inflammatory skin diseases.

## 1 Introduction

The skin is the largest organ in the human body and is mainly composed of three layers. The *epidermis* is most superficial layer and contains nerves and numerous specialized cells such as keratinocytes, melanocytes, Langerhans cells and Merkel cells (Orsmond et al., 2021). The dermis is located below the *epidermis* and is rich in fibrous extracellular matrix (ECM), containing fibroblasts and mast cells as well as interspersed resident immune cells like T cells, dendritic cells (DCs) and macrophages which participate in the immune response against pathogen infection (Orsmond et al., 2021; Naik, 2022). Moreover, abundant blood vessels and nerves are also found in the dermis. The deepest layer is the hypodermis, which is mostly composed of fatty tissue (Orsmond et al., 2021). In addition, the skin contains several appendages including hairs, hair follicles, sweat glands and sebaceous glands (Umar et al., 2018). These structures constitute a mechanical barrier to restrict water loss and protect the body from external environmental insults through physical and immune system functions.

In addition to its well-characterized mechanical barrier function, the skin is also the largest sensory organ of the human body, which can sense external cold, heat, touch, pressure pain, itch and other stimuli through receptors and primary sensory nerve endings (Middleton et al., 2021). Transient receptor potential (TRP) is a widely distributed protein in the peripheral and central nervous system, which is divided into seven subfamilies: TRPA (Ankyrin), TRPC (Canonical), TRPM (Melastatin), TRPN (NOMPC-like) TRPML (Mucolipin), TRPP (Polycystin) and TRPV (Vanilloid) (Venkatachalam and Montell, 2007; Samanta et al., 2018). TRPV1, the first protein found in TRP channels, is world-famous as its discoverer David Julius was awarded the 2021 Nobel Prize in Physiology or Medicine. Recent studies have revealed that TRPV1 is widely expressed in many cell types, including keratinocytes, sensory neurons and immune/inflammatory cells in the skin, attracting the attention of many researchers (Caterina and Pang, 2016; Bagood and Isseroff, 2021).

This review introduces the basic knowledge about the structure of the TRPV1 channel and discusses the expression of TRPV1 in the skin. Furthermore, the role of TRPV1 in skin aging and inflammatory skin diseases is explored.

## 2 Overall features of TRPV1 channels

**FIGURE 1:**
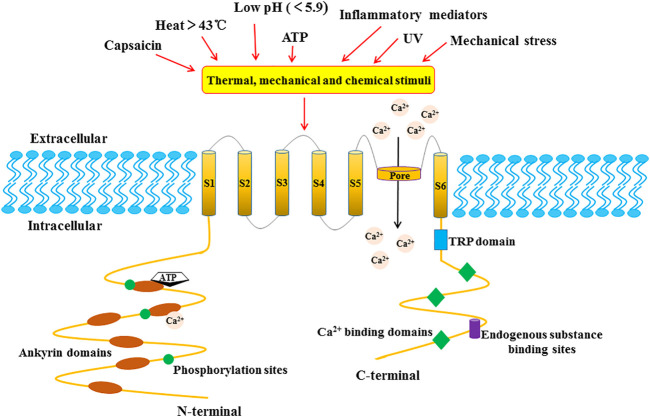
The structure of the TRPV1 channel subunit. TRPV1 is a homo-tetramer, and each subunit is composed of six-transmembrane domains (S1-S6) with a pore-forming hydrophobic group. The long N-terminal contains several phosphorylation sites and six ankyrin repeat domains. The C-terminal bears a TRP domain, multiple calmudulain binding domains and endogenous substance binding sites.

In the early 19th century, researchers pointed out that capsaicin, the main pungent component in “hot” chili pepper, caused a burning sensation and pain in mammalian mucosa or skin (Nagy et al., 2004). In 1997, Caterina et al. successfully cloned and isolated the first capsaicin-sensitive receptor from rat dorsal root ganglion and named it vanilloid receptor subtype 1 (VR1) (Caterina et al., 1997). The human TRPV1 protein composed of 838 amino acids is encoded by the TRPV1 gene with exons located at 17p13.2 (Hayes et al., 2000). The TRPV1 protein is a homo-tetramer, and each of its subunits consists of six-transmembrane domains (S1-S6), a long N-terminal region and a relatively short C-terminal region. Between the fifth and sixth transmembrane domains, a pore-forming hydrophobic span is considered to mediate the passage of ions (Caterina et al., 1997) (Figure 1). The N-terminal domain contains several phosphorylation sites and six ankyrin repeats to bind calmodulin and ATP (Lishko et al., 2007). Upon binding, these molecules modulate the sensitivity and function of TRPV1 (Phelps et al., 2010). On the other hand, the C-terminal domain bears a conserved TRP region, multiple calmodulin binding regions and endogenous substance binding sites, which can bind to protein kinase C (PKC), Ca^2+^/calmodulin-dependent protein kinaseⅡ(CaMK II), and phosphatidyl-inositol-4,5-bisphosphate (PIP_2_) (Garcia-Sanz et al., 2004).

TRPV1 is a multimodal nociceptor that is activated and/or allosterically modulated by various thermal, mechanical and chemical stimuli (Holzer, 2008). In addition to capsaicin, the thermal sensitivity of TRPV1 is controlled by other endogenous substances including ATP, bradykinin, nerve growth factor (NGF) (NatureChuang et al., 2001), inflammatory mediators such as arachidonic acid amides (Sinning et al., 2008) and prostaglandin (Zhong et al., 2021). Similarly, TRPV1 also is activated by other exogenous physical or chemical stimuli, such as camphor and resiniferatoxin (RTX), vanillatoxins 1–3 (tarantula), ginger, and ethanol (Holzer, 2008). Moreover, TRPV1 is activated under noxious heat conditions (≥43°C) (Loyd et al., 2012), acidosis (pH ≤ 5.9) (Kaszas et al., 2012), UV irradiation and mechanical stress (Inoue et al., 2009).

As a cation channel, TRPV1 shows similar permeability to monovalent cations such as Na^+^, K^+^, Li^+^, but it possesses moderate selectivity to divalent cations (Caterina et al., 1997). When TRPV1 is activated by capsaicin, the permeability of TRPV1 to ions is Ca^2+^ >Mg^2+^>Na^+^≈K^+^ (Caterina et al., 1997). The direct consequence of the TRPV1 activation is the inflow of extracellular Ca^2+^ into the cells and release of the intracellular Ca^2+^ pool, resulting in an increase in intracellular Ca^2+^ concentration, which mediates the basic activities of various cells, such as muscle contraction, neuronal activity, transmitter release, cell proliferation and apoptosis (Sappington et al., 2009). Notably, TRPV1 regulates body temperature and pain after activation. Studies have demonstrated that the activated TRPV1 promotes the release of substance P (SP) and calcitonin gene-related peptide (CGRP) (Brain and Grant, 2004). CGRP is a powerful vasodilator that significantly increases the blood flow of skin vessels, while SP is a short-term vasodilator that can significantly promote plasma extravasation. Both molecules can induce dermal vasodilation, local edema and other inflammatory reactions (Brain and Grant, 2004; Aubdool and Brain, 2011). In addition, SP mediated pruritus and influenced neurogenic inflammation *via* the neurokinin-1 receptor (NK-1R) and Mas-related G protein-coupled receptors (Mrgprs), which are located on both mast cells and on the cells of the dorsal horn of the spinal cord (Azimi et al., 2017). What’s more, the release of inflammatory mediators can mediate pain and hyperalgesia (Cavanaugh et al., 2009).

## 3 The expression and function of TRPV1 in skin

TRPV1 is considered a primary cellular sensor of thermal and chemical stimulation in the skin (Tominaga et al., 1998). In 2001, Denda et al. discovered the expression of TRPV1 in human epidermal keratinocytes (KC) and human epidermal immortalized keratinocyte line HaCaT cells (Denda et al., 2001). Subsequently, other studies demonstrated that heat, photoaging and natural aging could increase TRPV1 expression in human KC *ex vivo and in vivo* (Lee et al., 2008; Lee et al., 2009a). Theoretically, the TRPV1 agonist capsaicin evoked an influx of Ca^2+^ that was inhibited by the TRPV1 antagonist capsazepine or PAC-14028 in normal human keratinocytes (Inoue et al., 2002; Yun et al., 2011a). The dynamic balance of Ca^2+^ played an essential role in maintaining the stability of KC, and the increase of intracellular Ca^2+^ concentration promoted KC differentiation and inhibited KC proliferation (Thio et al., 1994). In addition, the activation of TRPV1 promoted the polarization and migration of KC (Li et al., 2012; Miyazaki et al., 2015).

Melanocytes are the cells primarily responsible for protecting against the harmful effects of UV irradiation on the skin. A study evaluated the TRPV1 immunoreactivity *in situ* in normal human skin, revealing that melanocytes were TRPV1 negative (Bodó et al., 2004). However, the researchers used immunocytochemistry and found positive TRPV1 expression in the cultured primary human melanocytes. Capsaicin could activate TRPV1 and increase intracellular Ca^2+^, but this did not affect melanogenesis (Choi et al., 2009). The contradictory results may arise from the low TRPV1 expression *in vivo,* which could not be detected using IHC. In contrast, a genome-wide transcriptome analysis of human epidermal melanocytes found the expression of TRPV1 (Haltaufderhyde and Oancea, 2014).

**FIGURE 2:**
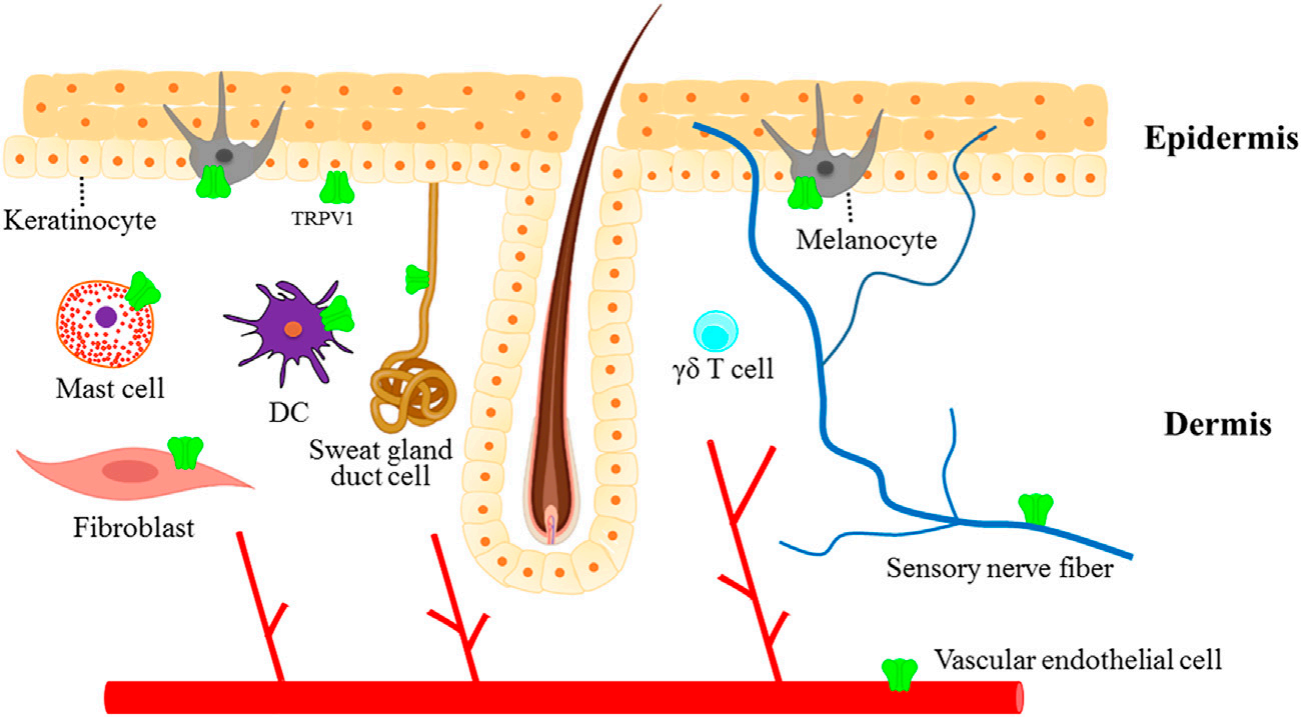
The expression of TRPV1 in the skin. TRPV1 channel is not only expressed in sensory nerve fibers innervating the skin but also in different non-neuronal cells, which contribute to maintaining normal skin physiology or play important roles in the pathogenesis of skin diseases.

Moreover, TRPV1 was also found to be expressed in skin fibroblasts (Lan et al., 2013), dermal vascular endothelial cells (Ständer et al., 2004), apocrine and exocrine sweat gland duct cells (Ständer et al., 2004), mast cells (Ständer et al., 2004) and dendritic cells (Basu and Srivastava, 2005) (Figure 2). TRPV1 activation by capsaicin triggered the maturation of immature DCs *in vitro* and the migration of dermal DCs to lymph nodes *in vivo* (Basu and Srivastava, 2005). In addition, it has been reported that TRPV1 is widely expressed on skin sensory nerve fibers (Nakagawa and Hiura, 2013). The activation of TRPV1^+^ sensory nerves with capsaicin produced pain, burning and itching and induced the release of CGRP and SP, mediating neurogenic inflammation, which eventually led to various skin diseases and pruritus (Nakagawa and Hiura, 2013). Another study revealed that TRPV1^+^ sensory neurons innervating the hair follicle promoted epithelial proliferation and hair follicle stem cell progeny migration after wounding (Martinez-Martinez et al., 2012). In human and mice, the stimulation of TRPV1 with capsaicin increased the insulin-like growth factor-I (IGF-1) levels and significantly promoted hair growth (Harada et al., 2007).

In summary, these findings improve the understanding of the complexity and plasticity of TRPV1 in the skin.

## 4 The role of TRPV1 in skin aging

**FIGURE 3:**
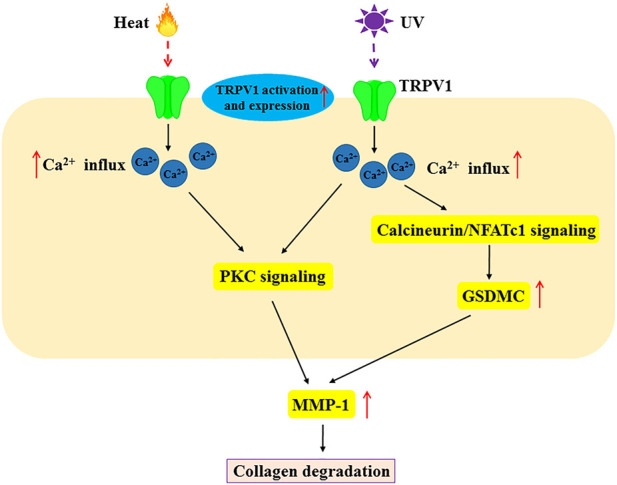
The molecular mechanism of TRPV1 in heat and UV-induced MMP-1 expression in skin aging. Heat and UV light not only activate TRPV1 but also increase its expression. Heat and UV induce MMP-1 expression *via* Ca^2+^ influx, PKC signaling and calcineurin/NFATc1 signaling. This scheme demonstrates the role of TRPV1 in skin aging by heat and UV. MMP-1, matrix metalloproteinase-1; PKC, protein Kinase C; NFATc1, nuclear factor of activated T-cells, cytoplasmic 1.

The aging of human skin is a complex process including intrinsic aging and photoaging (Lee et al., 2021). Intrinsic aging refers to chronological damage caused by slow, irreversible tissue degeneration, whereas photoaging is mainly induced by continuous UV exposure (Pourang et al., 2022). Wrinkles are one of the common characteristics of intrinsic aging and photoaging, which is caused by the degeneration of collagen and elastin fibers (Pedić et al., 2020). As well all known, collagen and elastin fibers are responsible for the strength and elasticity of the skin (Losquadro, 2017). Matrix metalloproteinase (MMP)-mediated collagen destruction contributes to wrinkle formation, laxity, and fragility during intrinsic skin aging and UV-induced photoaging (Lee et al., 2021). Some studies reported that TRPV1 and Ca^2+^ regulate the expression and activation of MMP-1 in UV-induced photoaging (Figure 3). UV irradiation increased TRPV1 mRNA and protein expression and activated TRPV1 to trigger Ca^2+^ influx, which in turn increased the MMP-1 mRNA and protein expression *via* Ca^2+^/calcineurin/NFATc1/GSDMC signaling in HaCaT cells (Lee et al., 2009a; Kusumaningrum et al., 2018). Additionally, MMP-1 expression induced by UV irradiation was mediated by TRPV1/Ca^2+^/PKC signaling in human keratinocytes (Lee et al., 2009b). A study has revealed that capsaicin boosted collagen synthesis in cultured dermal fibroblasts *via* extracellular signal-regulated kinases (Erk)/c-Jun-mediated MMP signaling. Moreover, the application of low-dose capsaicin on the shaved mouse dorsal skins alleviated the UV-induced reduction of collagen (Wu et al., 2022). In addition, baicalein suppressed MMP-1 expression by activating the TRPV1-Ca^2+^-ERK pathway in UVB-irradiated human dermal fibroblasts (Huang et al., 2019). Collectively, the various downstream signaling pathways of TRPV1/Ca^2+^ are involved in UV-induced skin aging. However, further research is required to determine if such mechanisms are present in chronological aging.

Thermal skin aging is caused by increased skin temperature during Sun exposure. Previous research has shown that acute heat shock induced TRPV1 expression (Matsumura and Ananthaswamy, 2004).

Another study reported increased expression of MMP-1 mRNA and protein in HaCaT cells and normal human epidermal keratinocytes following heat shock generated by infrared (IR) irradiation (Cho et al., 2009). Further study showed that heat-shock-induced MMP-1 expression was mediated by TRPV1 (Li et al., 2007). Expectedly, the inhibition of TRPV1 with TRPV1 inhibitors (capsazepine and ruthenium red) or knockdown of TRPV1 using RNA interference effectively suppressed heat shock-induced MMP-1 expression in HaCaT cells (Li et al., 2007). TRPV1-mediated Ca^2+^ influx and PKC signaling were necessary for heat shock-induced MMP-1 expression in HaCaT cells (Li et al., 2007). These findings suggest that TRPV1/Ca^2+^ signaling is involved in heat-induced MMP1 expression.

In summary, TRPV1/Ca^2+^ signaling is critical in skin aging. Therefore, the development of TRPV1-specific, non-toxic inhibitors may be an effective strategy for the prevention of skin aging.

## 5 TRPV1 and inflammatory skin diseases

The most common forms of skin diseases are inflammatory skin diseases such as psoriasis, atopic dermatitis (AD), rosacea, herpes zoster (HZ), allergic contact dermatitis (ACD) and prurigo nodularis (PN), which are caused by various internal and external infectious and non-infectious factors. Pruritus and erythema are the most common symptoms of skin diseases. Nevertheless, the specific mechanism is not fully understood. Currently, two signal pathways of pruritus have been identified, namely the histamine-dependent (histaminergic) signaling pathway and the protease-activated receptor-2 (PAR-2) signaling pathway (Song et al., 2018). These signaling pathways interact with mediators, such as amines (histamine, serotonin/5-HT, IL-6), peptides (bradykinin, SP, CGRP, neurotrophin) and phospholipid metabolites (cannabinoids, eicosanoids, platelet-activating factor), and result in the occurrence and development of pruritus and erythema (Song et al., 2018).

In recent years, a growing number of studies have investigated the potential role of TRPV1 in the pathogenesis of inflammatory skin diseases (Table 1). The following sections illustrate the mechanism and diverse functions in various inflammatory skin diseases and the potential clinical value of TRPV1.

**TABLE 1:** The role of TRPV1 in inflammatory skin diseases.

Diseases	Conclusion	Refs
Psoriasis	TRPV1 mRNA was overexpressed in PBMCs of patients	Ozcan et al. (2021)
	TRPV1 ablation reduced immune cells infiltration and the level of cytokines, such as IL-1β, IL-6, IL-23 and S100A8	Zhou et al. (2018)
	TRPV1^+^ sensory fibers ablation failed to control dermal DCs to produce IL-23, then decreasing IL-17 and IL-22 production by IL23R^+^ dermal γδ T cells and the subsequent reducing recruitment of inflammatory cells to the skin	Riol-Blanco et al. (2014)
	Capsaicin directly activated TRPV1, leading to Ca^2+^ influx, thus inhibiting the proliferation of keratinocytes and promoting the differentiation	Thio et al. (1994)
Atopic dermatitis (AD)	TRPV1 expression was upregulated in AD lesions	Steinhoff et al. (2003)
	TRPV1 is the common downstream signal of histamine dependent and PAR-2 pathway, involving in the generation of itch	Shim et al. (2007)
	Pruritus induced by IL-31 was mediated by directly activating IL-31RA on cutaneous TRPV1^+^ sensory nerve	Cevikbas et al. (2014)
	TRPV1 mediated itch-associated scratching and skin barrier dysfunction by regulating pruritogenic mediators (TSLP, IL-31 and SP) in DNFB-induced AD model	Tang et al. (2022)
	TRPV1 antagonist capsazepine and PAC-14028 could accelerate skin barrier function recovery	Denda et al. (2007), Yun et al. (2011a)
Rosacea	The expression of TRPV1 was significantly increased on non-neuronal cells in rosacea patients with erythema telangiectasia	Sulk et al. (2012)
	The activation of TRPV1 stimulated the sensory neurons C fibers to release vasoactive peptides (CGRP and SP), which contributes to inflammation	Aubdool and Brain (2011)
	The application of TRPV1 antagonist blocked the erythema and burning on human skin caused by external capsaicin cream	Chizh et al. (2007)
	TRPV1 antagonists have certain anti-depressant and anti-anxiety effects	de Moura et al. (2014)
Herpes zoster (HZ)	The protein and mRNA levels of TRPV1 were increased in the skin lesion of patient with HZ.	Han et al. (2016)
	The local pain response was related to the pro-inflammatory factor PGE2 TRPV1-mediated secreted by keratinocytes	
Allergic contact dermatitis (ACD)	Capsaicin-sensitive TRPV1^+^ neurons could overall act to downregulate the hyper-sensivity, possible by influencing the immune state of the skin	Banvolgyi et al. (2005)
Prurigo nodularis (PN)	In pruritic skin of PN, TRPV1 expression was highly increased in epidermal keratinocytes and nerve fibers	Ständer et al. (2004)
	Capsaicin treatment decreased the levels of SP and CGRP.	

PBMCs, peripheral blood mononuclear cells; PAR-2, protease-activated receptor-2; TSLP, thymic stromal lymphopoietin; SP, substance P; CGRP, calcitonin gene-related peptide; PGE2, prostaglandin E2.

### 5.1 TRPV1 and psoriasis

Psoriasis is a chronic, systemic immune-mediated disease characterized by erythematous, indurated, scaly, pruritic and often painful skin plaques that significantly impact patients’ quality of life (Vicic et al., 2021). Studies suggest that TRPV1 may be involved in the development of pruritus in psoriasis. A study found a positive correlation between TRPV1 overexpression and psoriasis itching (Nattkemper et al., 2018). The TrkA kinase inhibitor CT327 reduced pruritus by suppressing the NGF-TrkA-TRPV1 pathway in psoriatic patients (Roblin et al., 2015).

Further research demonstrated a close relationship between TRPV1 and the pathogenesis of psoriasis. Immune cells play a crucial role in the pathogenesis of psoriasis. A study reported increased TRPV1 mRNA expression in peripheral blood mononuclear cells (PBMCs) of patients with psoriasis (Ozcan et al., 2021). In the imiquimod (IMQ)-induced murine model of psoriasis-form dermatitis, TRPV1 gene knockout resulted in a reduction in the infiltration of CD45^+^ leukocytes, mast cells and CD3^+^ T, accompanied by a significant decrease in the expression levels of inflammatory cytokines (IL-1β, IL-6, IL-23 and S100A8) in the skin lesions of (61). In addition, upon selective pharmacological or genetic ablation of TRPV1^+^ sensory fibers, dermal dendritic cells failed to produce IL-23, resulting in decreased IL-17 and IL-22 expression by IL23R^+^ dermal γδ T cells. Subsequently, reduced recruitment of inflammatory cells to the skin was observed, eventually alleviating IMQ-induced IL-23-dependent psoriasis-like skin inflammation (Riol-Blanco et al., 2014). In preclinical models of psoriasis, resolving D3 (RvD3) derived from ω-3 fatty acids prevented the development of both psoriasis-form itch and skin inflammation with the concomitant decreased expression of CGRP in TRPV1^+^ neurons (Lee et al., 2020).

The balance between the proliferation and differentiation of the KCs is disrupted in psoriatic lesions, showing increased proliferation and impaired differentiation (Vicic et al., 2021). The concentration gradient of Ca^2+^ plays an important role in the differentiation of KCs. In the normal *epidermis*, the Ca^2+^ concentration is the highest in the granular layer, while that in the basal layer is the lowest (Lee and Lee, 2018). Interestingly, it has been demonstrated that the level of intracellular Ca^2+^ in the basal layer of psoriatic lesions was higher than extracellular Ca^2+^, indicating a disruption of the normal Ca^2+^ concentration gradient in psoriatic lesions (Cubillos and Norgauer, 2016). Furthermore, TRPV1-mediated Ca^2+^ flow was involved in the differentiation of KCs (Tsutsumi et al., 2011). Capsaicin directly activated TRPV1, leading to Ca^2+^ influx, thus inhibiting the proliferation of KCs and promoting the differentiation (Thio et al., 1994). Therefore, percutaneous delivery of capsaicin could significantly attenuate the psoriasis-form dermatitis symptom (Chan et al., 2021).

### 5.2 TRPV1 and atopic dermatitis (AD)

Atopic dermatitis (AD) is a chronic, genetically predisposed, complex inflammatory skin disease that is characterized by recurrent eczematous lesions, inflammation and intense itching (Nakahara et al., 2021). Intense pruritus affects patient behavior (scratching) and is associated with increased stress, anxiety, and other mood disorders, significantly worsening disease prognosis and quality of life (Nakahara et al., 2021). TRPV1 is a critical molecule in pruritus signaling in AD. TRPV1 has been shown to be upregulated in AD lesions, and its activation resulted in the release of mediators that promote inflammation and itching (Steinhoff et al., 2003). TRPV1 is involved in histamine-induced pruritus. Moreover, histamine-induced Ca^2+^ influx was attenuated by TRPV1 antagonists in rat dorsal root ganglion (DRG) neurons (Kim et al., 2004). A study has reported that human embryonic kidney 293 (HEK293) cells co-expressed TRPV1 and histamine receptors 1 (H1R) (Shim et al., 2007). In the presence of Ca^2+^, histamine binds to H1R to trigger the phospholipase A2 (PLA-2) and 12-lipoxygenase-dependent activation of TRPV1 on histamine-sensitive C-fibers, mediating itching (Shim et al., 2007). Thus, TRPV1-deficient mice demonstrated impaired scratching behavior in response to histamine injection (Shim et al., 2007; Imamachi et al., 2009). What’s more, a naturally occurring omega-9 fatty reduced itch evoked by histamine and cyclic phosphatidic acid (cPA) by inhibiting TRPV1 in peripheral neurons (Morales-Lázaro et al., 2016). To date, antihistamines have generally offered only marginal therapeutic benefits for AD. Thus, the combination of antihistamines and TPV1 antagonists provides a new strategy for the clinical treatment of AD.

Interleukin-31 (IL-31) is a major inflammatory marker whose level is positively correlated with the severity of AD (Datsi et al., 2021). TRPV1 and IL-31 receptor α (IL-31RA) are co-expressed in mouse and human dorsal root ganglion neurons. Pruritus induced by IL-31 is mediated by directly activating IL-31RA on the cutaneous TRPV1^+^ sensory nerve (Cevikbas et al., 2014). This evidence indicates a complex between TRPV1 and some mediators.

Furthermore, TRPV1 is involved in epidermal barrier homeostasis. TRPV1 was shown to mediate itch-associated scratching and skin barrier dysfunction by regulating pruritogenic mediators (including TSLP, IL-31 and SP) in a 2,4-dinitrofluorobenzene (DNFB)-induced AD model (Tang et al., 2022). In both mouse and human skin, capsaicin-mediated activation of TRPV1 retarded the skin barrier recovery. In contrast, the TRPV1 antagonists capsazepine and PAC-14028 could accelerate skin barrier function recovery by reducing TEWL (trans-epidermal water loss), promoting the reformation of the neutral lipid layer and reversing the changes in loricrin and filaggrin expression (Denda et al., 2007; Yun et al., 2011a). In addition, PAC-14028 attenuated histamine- and PAR-2-mediated pruritus in murine models of AD (Yun et al., 2011b). Phase II clinical trials to evaluate the efficacy of topical applications of PAC-14028 cream on AD have been completed. However, data from these trials were not available (ClinicalTrials.gov, 2017).

These findings suggest that TRPV1 is a potential therapeutic target for AD. In addition to traditional antipruritic therapy (antihistamines) to control itch symptoms, TRPV1 antagonists may be used as a new antipruritic drug to treat inflammatory skin diseases.

### 5.3 TRPV1 and rosacea

Rosacea is a chronic inflammatory cutaneous disorder characterized by repeated remission and recurrent episodes of flushing or transient erythema, mainly affecting the cheeks, nose, chin, and forehead (van Zuuren et al., 2021). Although the pathogenesis of rosacea is not fully understood, multiple factors are thought to be involved, such as genetics, immune factors, neurovascular dysregulation, microorganisms, and environmental factors (van Zuuren et al., 2021).

Substantial efforts have been made to elucidate the role of TRPV1 in the inflammatory stage of rosacea. A study has shown significantly increased expression of TRPV1 on non-neuronal cells in rosacea patients with erythema telangiectasia (Sulk et al., 2012). In general, the activation of TRPV1 stimulated the sensory neurons C fibers to release vasoactive peptides, such as CGRP and SP, leading to vasomotor dysfunction, and high neurovascular reactivity manifesting as paroxysmal flushing, persistent erythema and telangiectasia (Aubdool and Brain, 2011). Moreover, TRPV1 plays an essential role in heat sensitive regulation of inflammation. TRPV1-deficient mice demonstrated impaired response to high-temperature injury and acute thermal stimulation (Caterina et al., 2000). Chizh et al. reported that the application of TRPV1 antagonist blocked the erythema and burning caused by external capsaicin cream on human skin (Chizh et al., 2007). Some studies have revealed that TRPV1 antagonists have certain antidepressant and antianxiety effects, which may be beneficial to rosacea patients as they often suffer from psychological stress (de Moura et al., 2014). Therefore, TRPV1 may be a new therapeutic target for rosacea.

### 5.4 TRPV1 and other inflammatory skin diseases

Herpes zoster (HZ), also known as shingles, is a skin infection disease characterized by debilitating pain caused by the reactivation of varicella-zoster virus (VZV) (Rosamilia, 2020). Elevated protein and mRNA levels of TRPV1 were observed in the skin lesions of HZ patients. Interestingly, the enhanced mRNA and protein expression level of prostaglandin E2 (PGE2) were also detected in skin lesions, suggesting a correlation between TRPV1 and pain in HZ patients. The local pain response may be related to the pro-inflammatory factor PGE2 secreted by TRPV1-mediated keratinocytes (Han et al., 2016).

Allergic contact dermatitis (ACD) is a common inflammatory skin disease caused by exposure to contact allergens (Olusegun and Martincigh, 2021). Banvolgyi et al. successfully depleted the TRPV1-mediated sensory component from the capsaicin-sensitive neurons by systemic resiniferatoxin (RTX) pretreatment or genetic deletion, resulting in an enhanced inflammatory response. This enhancement indicated that capsaicin-sensitive neurons expressing TRPV1 can downregulate hypersensitivity, possibly by influencing the immune state of the skin (Banvolgyi et al., 2005).

Prurigo nodularis (PN) is an extremely severe pruritic skin disease characterized by intensely pruritic, hyperkeratotic nodules (Williams et al., 2021). A significant increase in TRPV1 expression was observed in epidermal KCs and nerve fibers of the pruritic skin of PN, which was normalized after capsaicin application (Ständer et al., 2004). During repeated application of capsaicin therapy, a reduction of neuropeptides (such as SP and CGRP) from sensory nerve fibers was observed due to the desensitization of nerve fibers. After capsaicin treatment was stopped, neuropeptides re-accumulated in cutaneous nerves, which suggests that TRPV1 plays an important role in pruritus and neurogenic inflammation (Ständer et al., 2004).

## 6 Conclusion and future perspectives

Since TRPV1 was discovered in the skin, it has gradually revealed the important role in the physiological and pathological skin. This review focuses on the structure and biological characteristics of the TRPV1 channel and its expression in various skin cells, such as cutaneous sensory nerve fibers, mast cells, epidermal keratinocytes, immune cells, dermal blood vessels, the inner root sheet and the infundibulum of hair follicles, differentiated sebocytes, sweat gland ducts, and the secretory portion of eccrine sweat glands. Notably, TRPV1 exerts different functions in various cell types. Furthermore, a great deal of studies have demonstrated the involvement of TRPV1 in skin aging and skin inflammation through different mechanisms.

The activation or blockade of TRPV1 is a promising therapeutic strategy in the treatment of chronic inflammatory skin diseases. An in-depth understanding of the biological function of TRPV1 and its role in the pathogenesis of chronic inflammatory skin diseases will allow the development of specific TRPV1 agonists or antagonists for the treatment of chronic inflammatory skin diseases.
